# Hydrothermal Synthesis of Cr_2_Se_3_ Hexagons for Sensitive and Low-level Detection of 4-Nitrophenol in Water

**DOI:** 10.1038/s41598-018-23243-3

**Published:** 2018-03-19

**Authors:** Sukanya Ramaraj, Sakthivel Mani, Shen-Ming Chen, Selvakumar Palanisamy, Vijayalakshmi Velusamy, James M. Hall, Tse-Wei Chen, Tien-Wen Tseng

**Affiliations:** 1 Electroanalysis and Bioelectrochemistry Lab, Department of Chemical Engineering and Biotechnology, National Taipei University of Technology, Taipei, Republic of China; 2 Division of Electrical and Electronic Engineering, School of Engineering, Manchester Metropolitan University, Manchester, UK

## Abstract

We report a simple hydrothermal method used for the synthesis of Cr_2_Se_3_ hexagons (h-Cr_2_Se_3_) and its application towards electrochemical sensing of 4-nitrophenol (4-NP). The formation of h-Cr_2_Se_3_ was confirmed by using scanning electron microscopy, energy dispersive X-ray spectroscopy, X-ray diffraction, and X-ray photoelectron spectroscopy. The electrochemical activity of the h-Cr_2_Se_3_ modified screen-printed carbon electrode (SPCE) towards 4-NP was studied using cyclic voltammetry (CV) and amperometric i-t techniques. Typically, the obtained results were compared with those for a bare SPCE. The CV result clearly reveals that h-Cr_2_Se_3_ modified SPCE has higher catalytic activity towards reduction of 4-NP than bare SPCE. Hence, h-Cr_2_Se_3_ modified SPCE was concluded as a viable sensor for sensitive determination of 4-NP. Under optimized conditions, h-Cr_2_Se_3_ modified SPCE demonstrates the excellent capacity to detect the 4-NP in a linear range from 0.05 µM to 908.0 µM. The LOD and sensitivity in detection of 4-NP were determined at 0.01 µM and 1.24 µAµM^−1^ cm^−2^ respectively. The sensor is highly selective and stable and shows reproducible recovery of 4-NP in domestic supply and river water samples.

## Introduction

The synthesis of novel materials has received significant interest across many disciplines including analytical chemistry. In particular, the synthesis of transition metal chalcogenides has been the subject of considerable attention and derived materials have been widely utilized in supercapacitors^1^, solar cells^2^, batteries^3^, and sensors^4^ due to their high energy density, long cycling stability, and excellent electrochemical and charge transfer properties^5–7^. Accordingly, transition metal chalcogenides such as metal sulfide, selenide, telluride, nitride, boride, and phosphide have also been widely prepared and employed in energy and sensor applications^8–11^. Given their superior electronic characteristics, selenide based chalcogenides are recognized as superior to other metal chalcogenides. Recently, many chalcogenides selenides such as Ni, Co, Fe and Mo selenides have been reported to date. Metal selenides are typically prepared by using chemical bath deposition^12^, chemical vapor deposition^13^, electrodeposition^14^, simple chemical synthesis^15^, and hydrothermal techniques^16^. Among these, hydrothermal techniques are shown to generate metal selenides of varying structure and high crystallinity^17^. In the present work, we have synthesized Cr_2_Se_3_ hexagon (h-Cr_2_Se_3_) using a simple hydrothermal method for the first time.

4-nitrophenol (4-NP) is well-known phenolic compound that has been widely used in the industrial manufacture of products from pesticides and fungicides to paracetamol and dyes^18^. However, 4-NP is also considered as a major water pollutant with serious health implications for both humans and animals^19^. Reliable and sensitive detection of 4-NP in water samples is therefore essential to the treatment and provision of safe water supplies. To date, various analytical methods such as mass spectrometry^20^, high performance liquid chromatography^21^, spectrophotometry^22^, flow injection analysis^23^, and electrochemical methods^24–29^ have all been applied to the sensitive determination of 4-NP concentrations in water samples. However, the determination of 4-NP levels by electrochemical techniques is demonstrated to be less complex and less expensive than other reported methods^25,26^. Owing to their increased surface area, high conductivity, and unique physical and chemical properties, chemically modified electrodes have been widely used for electrochemical determination of 4-NP in recent years. Accordingly, electrodes modified with boron doped diamond film^27^, Hg (mercury hanging drop)^24^, and its amalgam (Ag, Cu, Au, Bi, Sn, or Zn with liquid mercury)^28,29^ have been widely applied in the determination of 4-NP. In addition, the composites of carbon micro/nano materials^30,31^, metal oxides^32^ and conducting polymers^33^ have also been routinely used in the sensitive detection of 4-NP. The main goal of the present work is to fabricate a sensitive and selective sensor for detection of 4-NP by using h-Cr_2_Se_3_ as a model active electrode material. It is noted that the application of metal selenides in electrochemical determination of 4-NP is limited. However, given its high conductivity and high relative surface area, the application of h-Cr_2_Se_3_ modified electrodes in the determination of 4-NP may demonstrate significant advantages in terms of high sensitivity, a wide linear range, and reduced detection limit (LOD).

## Results and Discussion

### Characterizations of h-Cr_2_Se_3_

**Figure 1:**
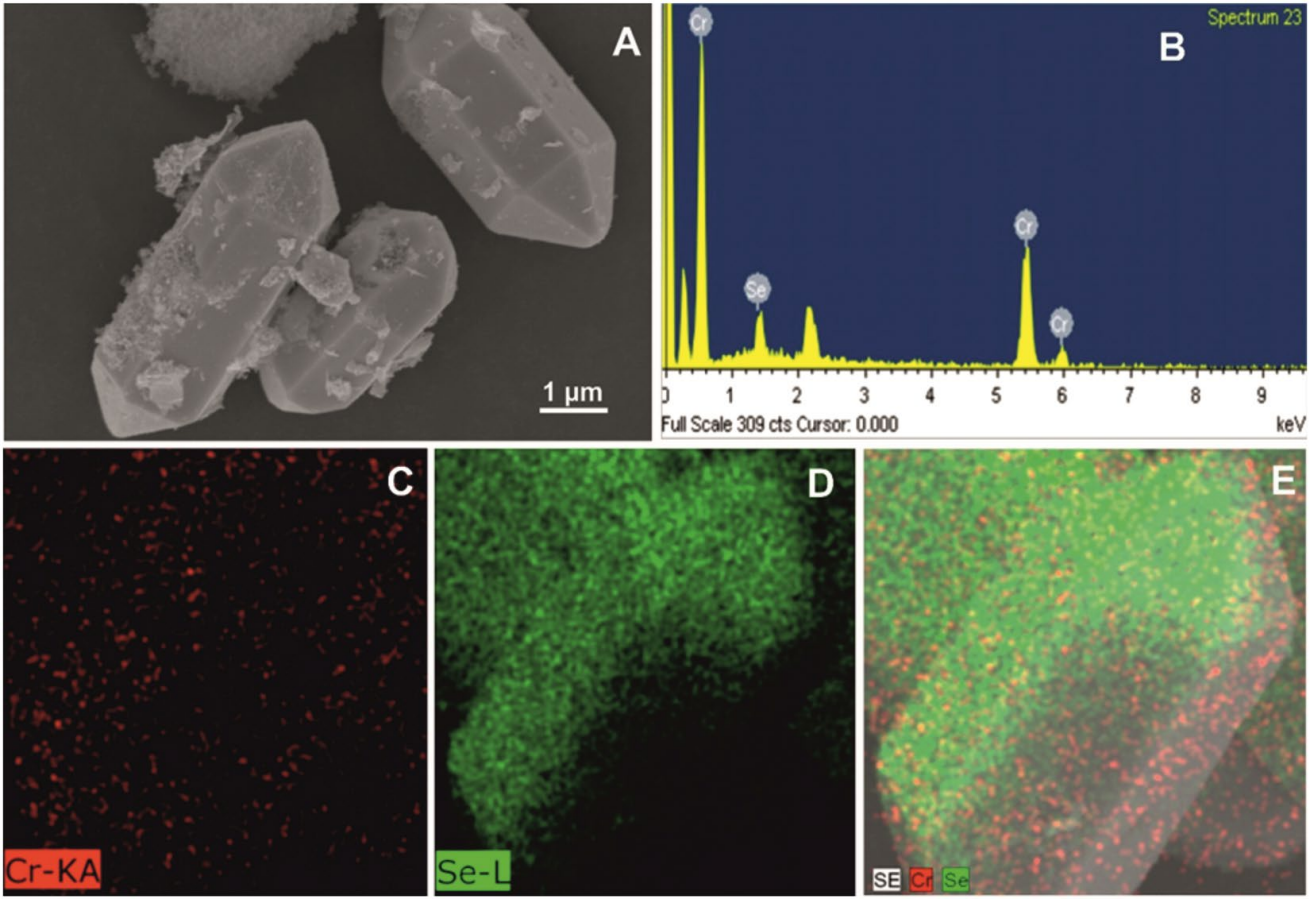
(**A**) SEM image of h-Cr_2_Se_3_, and EDX (**B**) and elemental mapping (**C**–**E**) of h-Cr_2_Se_3_.

**Figure 2:**
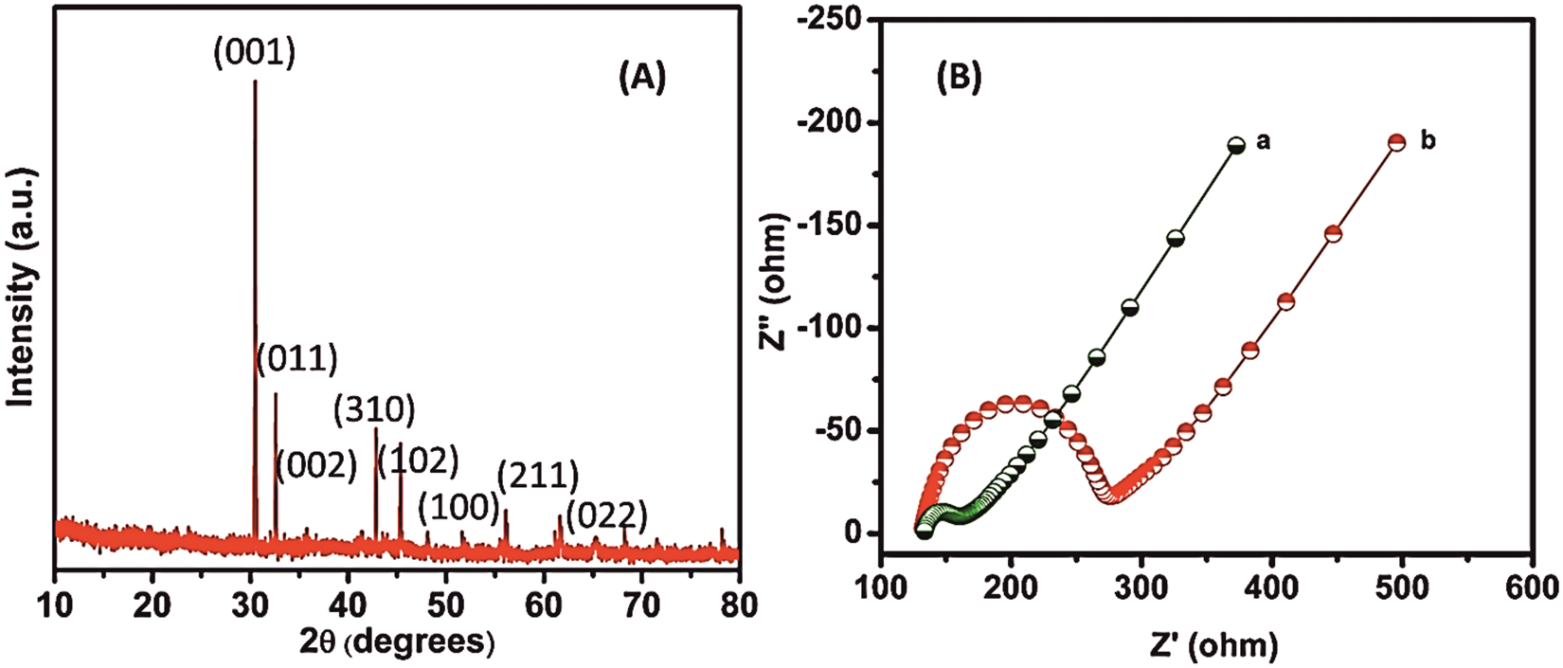
(**A**) XRD pattern of as-synthesized h-Cr_2_Se_3_. (**B**) EIS Nyquist curve of (a) h-Cr_2_Se_3_/SPCE and (b) bare SPCE in 5 mM of [Fe(CN)6]^3−/4−^ containing 0.1 M of KCl in the frequency range from 0.1 Hz to 100 kHz.

The surface morphology of as-synthesized h-Cr_2_Se_3_ was examined by SEM. The SEM image of as prepared h-Cr_2_Se_3_ is shown in Fig. 1A, and clearly demonstrates the hexagonal structure of Cr_2_Se_3_. Figure 1B shows the EDX of as-synthesized h-Cr_2_Se_3_ and confirms the presence of Cr and Se in h-Cr_2_Se_3_. In addition, the elemental mapping of h-Cr_2_Se_3_ (Fig. 1C,D) reveals the uniform distribution of Cr and Se in h-Cr_2_Se_3_. The results confirmed the formation of h-Cr_2_Se_3_. We have used XRD to confirm the crystalline characteristics of h-Cr_2_O_3_. Figure 2A shows the XRD pattern of h-Cr_2_Se_3_, and all major diffraction peaks obtained concur with the standard data for Cr_2_Se_3_ (JCPDS NO. 98-010-6520) with hexagonal structure (space group R-3, NO. 148). The cell parameters of Cr_2_Se_3_ hexagon are a = 6.25 Å, b = 6.25 Å, c = 17.28 Å. In this XRD pattern, the diffraction peaks of h-Cr_2_Se_3_ are located at 30.48°, 32.58°, 42.83°, 45.33°, 51.73°, 56.06°, 61.63°, and 68.31° for corresponding lattice plane of (001), (011), (002), (310), (102), (100), (211), and (022). The sharp peaks highlight the high crystalline purity of h-Cr_2_Se_3_. Accordingly, the reported method is suited to the preparation of a metal chalcogenide with high purity.

EIS is a powerful tool to investigate electron charge transfer processes at the interface between electrode and electrolyte related to double layer capacitance (c_dl_), solution resistance (R_s_), Warburg impendence (Z_W_), and charge transfer resistance (R_ct_)^34^. In general, the semicircle region at higher frequency region and its diameter are ascribed to charge transfer resistance (R_ct_). Figure 2B shows the EIS profile of bare SPCE and h-Cr_2_Se_3_/SPCE in 5 mM [Fe(CN)6]^3−/4−^ containing 0.1 M of KCl in the frequency range from 0.1 Hz to 100 kHz. The R_ct_ values for bare SPCE and h-Cr_2_Se_3_/SPCE were calculated as 136.8 and 28.45 Ω, respectively. This confirms h-Cr_2_Se_3_/SPCE has a higher electron transfer ability than the bare SPCE.

Cyclic voltammetry (CV) was used to investigate the electron transfer ability of bare SPCE and h-Cr_2_Se_3_/SPCE, with electrochemical experiments employing an electrolyte of 5 mM of [Fe(CN)6]^3−/4−^ contain 0.1 M of KCl, and at a scan rate of 50 mV/s. The obtained voltammetry data are shown in Figure. S1A. When compared to the un-modified SPCE, the h-Cr_2_Se_3_/SPCE displays clearly enhanced oxidation and reduction peak current, and a peak-to-peak separation of 0.18 V (Figure. S1B) 90 mV lower than observed for the un-modified SPCE. This result demonstrates the enhanced electron transfer capacity and reversibility because of h-Cr_2_Se_3_/SPCE modification.

**Figure 3:**
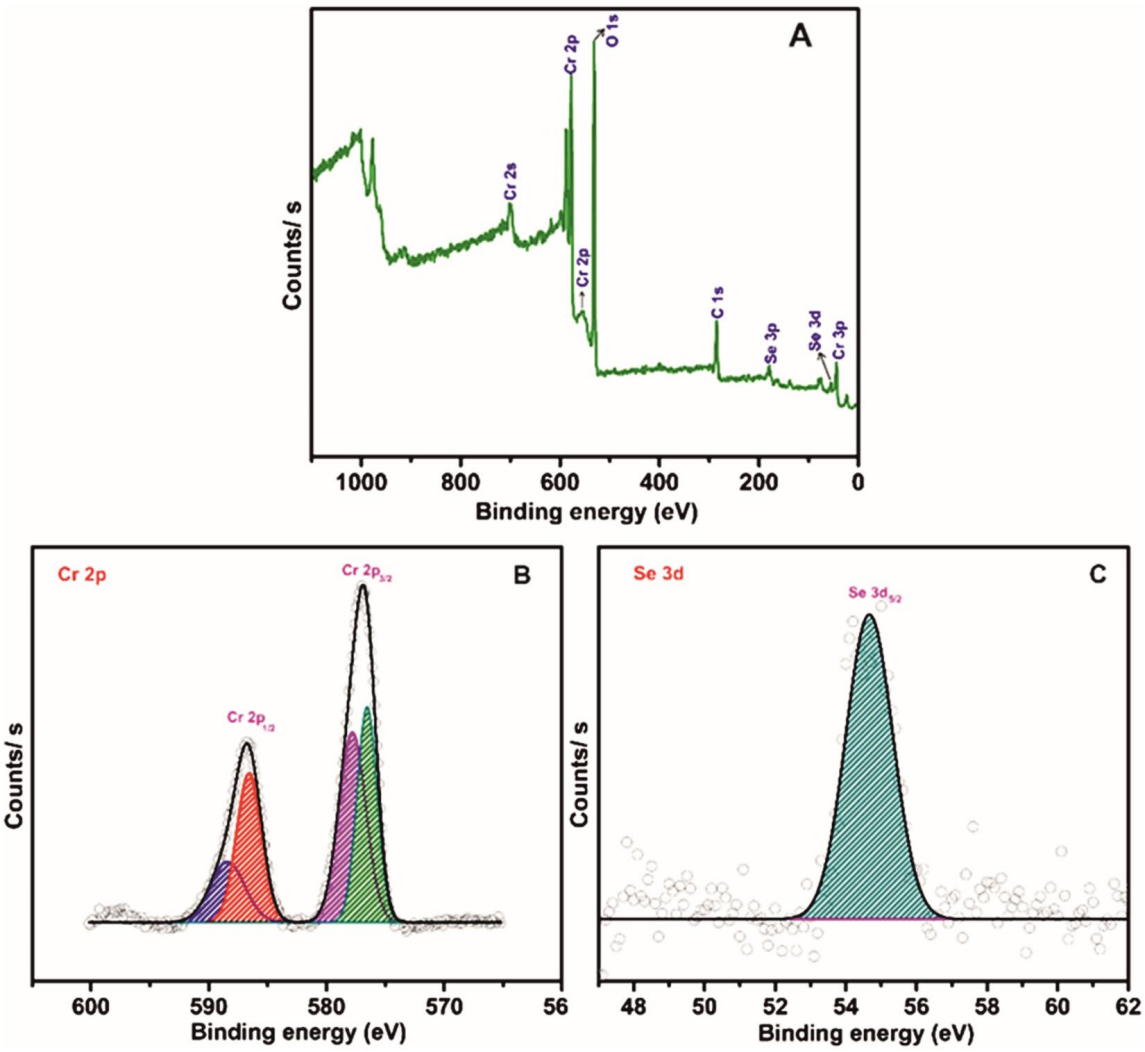
(**A**) Wide scan XPS spectra of h-Cr_2_Se_3_. (**B**) XPS spectra of Cr 2p, and (**C**) Se 3d.

The surface chemical state of Cr and Se elements in h-Cr_2_Se_3_ was probed by using XPS analysis as shown in Fig. 3. The survey spectrum of h-Cr_2_Se_3_ (Fig. 3A) clearly exhibits the major peaks of Cr, Se, O, and C. This reveals that the sample h-Cr_2_Se_3_ mainly contains the Cr and Se elements at near to surface range while the presence of carbon (as the reference) and oxygen is assigned to the surface adsorption of hydrocarbon contaminants and moisture. Figure 3B shows the XPS spectra of Cr 2p with two energy band at 586.8 and 576.9 eV for corresponding Cr 2p_1/2_ and Cr 2p_3/2_ states respectively, where the peak binding energy separation was determined as approximately 9.9 eV. The Se 3d spectrum in Fig. 3C shows the band for Se 3d_5/2_ at 54.6 eV, which confirms the presence of the metal selenium bond. The XPS analysis evidently proves the chemical states of both Cr and Se in the h-Cr_2_Se_3_, in agreement with the published literature^35,36^.

### Electrochemical behavior of 4-_NP_

**Figure 4:**
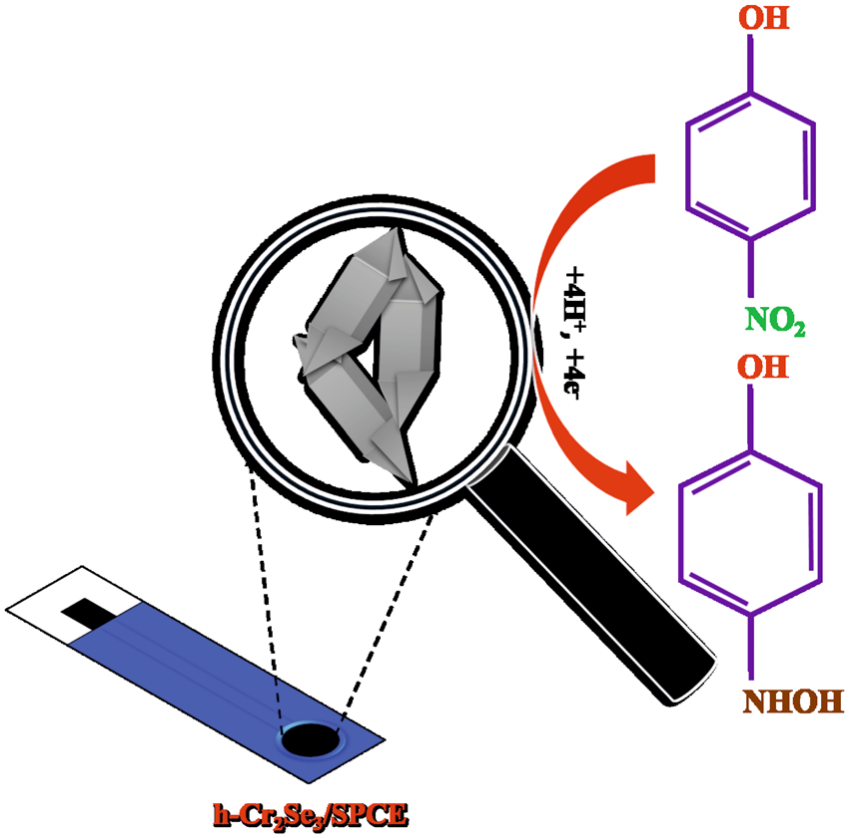
Schematic representation for the eelectrochemical reduction of 4-NP at h-Cr_2_Se_3_ modified SPCE.

**Figure 5:**
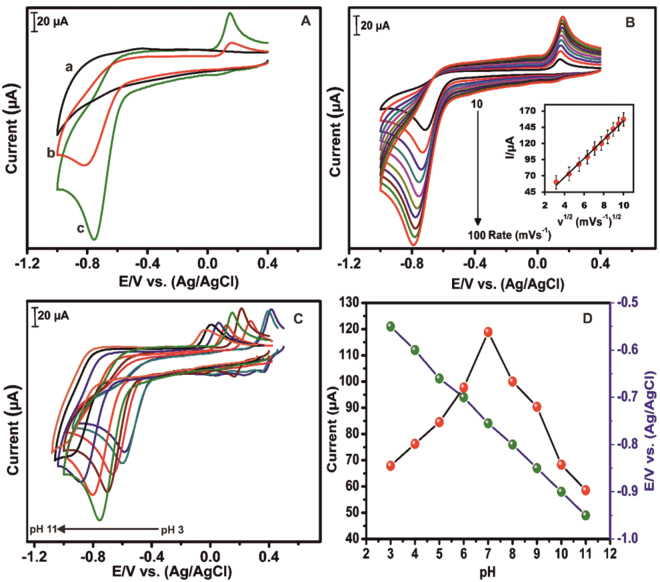
(**A**) CV response of h-Cr_2_Se_3_/SPCE in the absence (a) and presence (c) of 476 µM 4-NP at pH 7 at scan rate of 50 mVs^−1^. At same conditions, CV response of bare SPCE in the presence of 476 µM 4-NP at pH 7. (**B**) CVs obtained for h-Cr_2_Se_3_ modified SPCE in 476 µM of 4-NP at pH 7 against an increasing scan rate from 10 to 100 mVs^−1^. The inset figure shows the relationship between the square root of scan rate and the resulting current response. (**C**) CV response of h-Cr_2_Se_3_ modified SPCE in 476 µM 4-NP for pH values from pH 3 to pH 11 at a scan rate of 50 mVs^−1^. (**D**) Corresponding plot of pH vs. E_pc_ and pH vs. I_pc_.

The schematic representation for the electrochemical reduction of 4-NP at h-Cr_2_Se_3_ modified SPCE is shown in Fig. 4. The electrocatalytic activity of h-Cr_2_Se_3_/SPCE and bare SPCE towards the detection of 4-NP was studied by CV. Figure 5A shows the CV response of h-Cr_2_Se_3_/SPCE and bare SPCE in presence and absence of 4-NP at pH 7. The h-Cr_2_Se_3_/SPCE exhibits a sharp reduction peak at −0.75 V in the presence of 476 µM 4-NP (curve c), which is due to the direct reduction of 4-NP into hydroxylaminophenol^37^ as in Fig. 4. In addition, a quasi-reversible anodic peak was observed at 0.14 V due to oxidation of hydroxylaminophenol into 4-nitrosophenol. It should be noted that no such peaks were obseved at h-Cr_2_Se_3_/SPCE in the absence of 4-NP (curve a). It is notable that the reduction peak potential of 4-NP was 70 mV lower at h-Cr_2_Se_3_ modified SPCE when compared to the response observed for bare SPCE (curve b). In addition, the observed reduction peak current of 4-NP at h-Cr_2_Se_3_/SPCE was 2-fold higher than bare SPCE. The unique properties of h-Cr_2_Se_3_ on SPCE results in enhanced sensitivity and low potential detection for 4-NP. Hence, h-Cr_2_Se_3_ modified SPCE can be used for sensitive and lower potential detection of 4-NP.

Generally, the electrochemical behaviour of the modified electrodes is greatly controlled by the effect of applied scan rate. Therefore, the effect of scan rate on the surface of h- Cr_2_Se_3_/SPCE towards detection of 476 µM 4-NP was studied by CV. Figure 5B shows the CV response of h-Cr_2_Se_3_/SPCE at pH 7 containing 476 µM 4-NP at increasing scan rates. The reduction peak current of 4-NP increases with the scan rate from 10 to 100 mVs^−1^. In addition, the oxidation peak current of 4-nitrosophenol also increases with increasing the scan rates. As shown in Fig. 5B inset, the reduction current of 4-NP has a linear relationship with the squre root of scan rates. The correlation coeffecient (R^2^) was found to be 0.995. The result implies that the overall electrochemical reduction reaction of 4-NP at h-Cr_2_Se_3_/SPCE is a typical diffusion controlled process^38^.

The electrocatalytic ability of the modified electrode towards 4-NP was studied at different pH, since electrochemical activity of the modified electrode can be easily affected by pH. Figure 5C shows the CV response of h-Cr_2_Se_3_/SPCE in 476 µM 4-NP across a range of pH values from pH 3 to pH1 at a scan rate of 50 mV/s. The h-Cr_2_Se_3_ modified SPCE shows a sharp reduction peak current response in the presence of 4-NP at each pH, with the maximum reduction peak current response was observed at pH 7 (Fig. 5D). This may be due to the high activity of h-Cr_2_Se_3_ modified SPCE in pH 7 than other pHs. Accordingly, pH 7 was selected as optimal for further electrochemical studies. Figure 5D also illustrates the linear relationship between pH and current peak potential. The linear regression was calculated as E (V) = −0.507 − 0.399 pH with an R^2^ value of 0.9977. The negative sign indicates the proton to be directly involved in the electrochemical reduction of 4-NP, and such findings are consistent with previously reported results^38^.

### Determination of 4-NP

The electroctalytic ability of h-Cr_2_Se_3_ modified SPCE towards the detection of different concetration of 4-NP was investigated by CV. Figure. S2 shows the CV response of h-Cr_2_Se_3_ modified SPCE in the absence (a) and presence of 100 µM (b), 380 µM (c), 650 µM (d) and 909 µM 4-NP at pH 7, and at scan rate of 50 mV/s. In the absence of 4-NP, the h-Cr_2_Se_3_ modified SPCE did not show any apparent electrochemical response at pH 7, while a significant reduction peak current was observed at h-Cr_2_Se_3_ modified SPCE in the presence of 100 µM of 4-NP. The reduction peak current increases with increasing concentrations of 4-NP at pH 7 (c-e) and indicates a high electro-reduction capacity of h-Cr_2_Se_3_ modified SPCE towards 4-NP.

**Figure 6:**
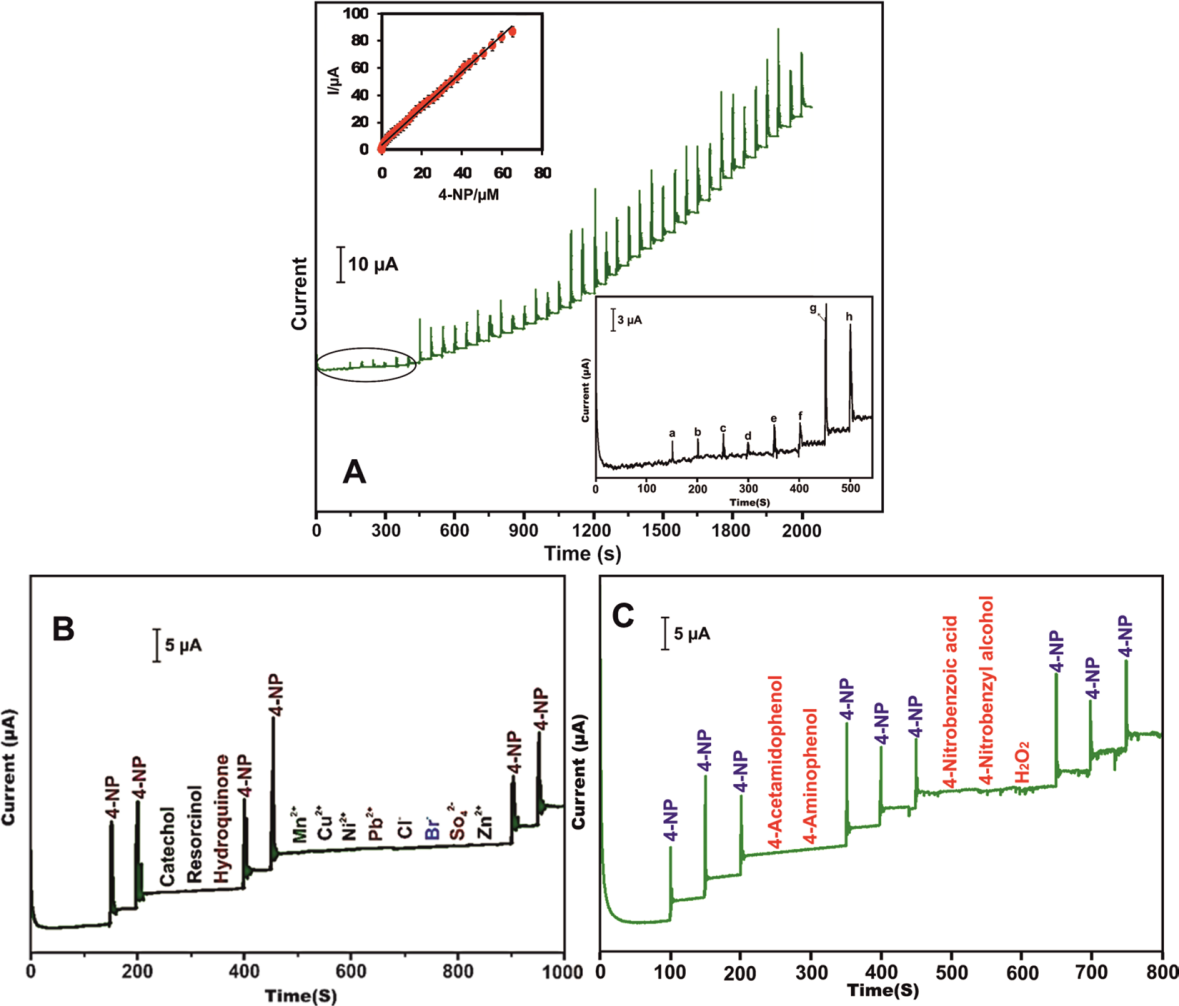
(**A**) Amperometric i-t response of h-Cr_2_Se_3_ modified SPCE for addition of different concentration of 4-NP into the constantly stirred pH 7. Inset is calibration plot for current response vs. [4-NP], and an enlarged view of amperometric response of h-Cr_2_Se_3_ modified SPCE for addition of 0.05 µM (a), 0.1 µM (b), 0.2 µM (c), 0.7 µM (d), 1.5 µM (e), 2.5 µM (f), 4 µM (g) and 5.7 µM (h) 4-NP at the working potential of −0.73 V. (**B**) Amperometric *i-t* response of h-Cr_2_Se_3_ modified SPCE for addition of 10 µM 4-NP and 500 µM additions of dihydroxybenzene isomers and metal ions at pH 7 with the working potential of −0.73 V. (**C**) At similar conditions, amperometric *i-t* response of h-Cr_2_Se_3_ modified SPCE for addition of 10 µM of 4-NP and 500 µM additions of phenolic and nitro compounds.

Amperometric *i-t* method was used to determine the 4-NP using h-Cr_2_Se_3_ modified SPCE. Under optimized conditions, the h-Cr_2_Se_3_ modified SPCE is used for the detection of 4-NP in constantly stirred pH 7 with the working potential of −0.73 V. As shown in Fig. 6A, a sharp amperometric response was observed for each addition of different concetration of 4-NP (0.05–958.0 µM) at pH 7. The sensor also shows a stable response for addition of 0.05 µM (a), 0.1 µM (b), 0.2 µM (c), 0.7 µM (d), 1.5 µM (e), 2.5 µM (f), 4 µM (g) and 5.7 µM (h) 4-NP into the constantly stirred PBS (Fig. 6A lower inset). The response time of the sensor was calculated as 4 s and reveals the fast electrocatalytic reduction of 4-NP by h-Cr_2_Se_3_ modified SPCE. In addition, the amperometric response current of 4-NP was linear over concentrations ranging from 0.05 µM to 908.0 µM (Fig. 6A upper inset). The linear equation for the calibration plot is I (μA) = 0.15 + 4.71 *C* (µM) and the R^2^ is 0.9967. The sensitivity of the sensor is 1.24 µAµM^−1^ cm^−2^ as calculated from the slope of the calibration plot/electrochemically active surface area (0.12 cm^2^) of the h-Cr_2_Se_3_ modified SPCE. The detection limit (LOD) of the sensor was estimated as 0.01 µM based on 3 ∗ standard deviation of the blank response/slope of the calibration plot (0.15), where the blank response currents are 0.0124, 0.0131 and 0.0121 µA (Fig. 6A lower inset). To further verfiry the advantages of the Cr_2_Se_3_ modified SPCE for 4-NP sensor applications, the LOD, senstivity and linear response range of the Cr_2_Se_3_ modified SPCE sensor was compared with previously reported modified electrodes. The comparative results are shown in Table 1, and clearly show the h-Cr_2_Se_3_ modified SPCE has lower LOD, wider linear response range and higher senstivity for the detection of 4-NP than previously reported modified electrodes. For instance, the LOD of h-Cr_2_Se_3_ modified SPCE (0.01 µM) was lower than nano- Au^25^ (8 µM), graphene-chitosan^26^ (0.08 µM), chitosan/ZnO nano needles^30^ (0.23 µM), cyclodextrin-reduced graphene oxide^33^ (0.05 µM), activated carbon^31^ (0.16 µM), nano-Cu_2_O^32^ (0.5 µM), Ag nanoparticles^38^ (0.015 µM), carbon dot^39^ (0.028 µM), ZnO^40^ (0.029 µM) and hydroxyapatite nano powder^41^ (0.6 µM) modified electrodes. In addition, the senstivity and linear response of the sensor was more comparable with the previously reported sensors^25,26,30–33,38–42^.

**Table 1:** Comparison of analytical performance of h-Cr_2_Se_3_ modified SPCE with previously reported modified electrodes for determination of 4-NP. Abbreviations LOD – limit of detection; GCE – glassy carbon electrode; CV – cyclic voltammetry; Gr – graphene; CHI – chitosan; ABPE – acetylene black paste electrode; LSV – linear sweep voltammetry; NDs – nano needles; DPV – differential pulse voltammetry; CD – cyclodextrin; RGO – reduced graphene oxide; AC –activated carbon; PDPA – poly(diphenylamine); HA-NP – hydroxyapatite nano powder.

**Electrode material**	**Method of detection**	**LOD (µM)**	**Linear range (µM)**	**Sensitivity (µA µM** ^**−1**^ **cm** ^**−2**^ **)**	**Ref**
Nano-Au/GCE	CV	8	10–30	—	^25^
Gr-CHI/ABPE	LSV	0.08	0.1–80	—	^26^
CHT/ZnONDs/GCE	DPV	0.23	0.5–400.6	0.64	^30^
AC-modified GCE	LSV	0.16	1–500	5.810	^31^
Nano-Cu_2_O/GCE	DPV	0.5	1.0–400	—	^32^
CD-RGO/GCE	DPV	0.05	1–10	0.64	^33^
AgNPs/GCE	DPV	0.015	100–350	2.58	^38^
Carbon dot	Fluorescence	0.028	0.1–50	—	^39^
MWNTs-PDPA/GCE	Amperometry	—	8.9–1430	0.63	^40^
ZnO/GCE	DPV	0.209	10–40	0.04	^41^
HA-NP/GCE	Amperometry	0.6	1.0–300	—	^42^
h-Cr_2_Se_3_/SPCE	Amperometry	0.01	0.05–908	1.24	This work

#### Selectivity studies

The selectivity of the h-Cr_2_Se_3_ modified SPCE towards detection of 4-NP was investigated by amperometric method. Figure 6B shows the amperometric response of h-Cr_2_Se_3_ modified SPCE for the addition of 10 µM of 4-NP and 500 µM additions of catechol, resorcinol, hydroquinone, Mn^2+^, Cu^2+^, Ni^2+^, Pb^2+^, Cl^−^, Br^−^, SO_4_^2−^ and Zn^2+^ at pH 7 with an applied potential of –0.74 V. A well-defined and sharp amperometric response was observed for the addition of 4-NP. The previously discussed electroactive interferences did not show any response on h-Cr_2_Se_3_ modified SPCE. Accordingly, the results clearly reveal that h-Cr_2_Se_3_ modified SPCE is highly selective for detection of 4-NP in the presence of phenolic compounds and metal ions. We have also tested the selectivity of the sensor in the presence of electrochemically reducible compounds such as 4-acetamidophenol, 4-aminophenol, 4-nitrobenzoic acid, 4-nitrobenzyl alcohol and H_2_O_2_. The selectivity studies were performed in the presence of 50-fold additions of the aforementioned electrochemically reducible compounds by amperometry and the results are shown in Fig. 6C. It can be seen that the Cr_2_Se_3_ modified SPCE shows a stable amperometric response for the addition of 10 µM of 4-NP and 500 µM additions of electrochemically reducible compounds did not result in any measureable response. Hence, the modified sensor can be used for the selective detection of 4-NP in the presence of electrochemically reducible compounds and metal ions.

#### Stability, repeatability, and reproducibility of the sensor

Stability, repeatability, and reproducibility are critical to utilization of the sensor in real time applications. Fig. S3 shows the operational stability of h-Cr_2_Se_3_ modified SPCE for the addition of 10 µM of 4-NP into the constantly stirred electrolyte at pH 7, and the background current response up to 1200 s. The amperometric profile of h-Cr_2_Se_3_ modified SPCE clearly shows that the sensor retains 97.3% of its initial current response after 1200 s. This result demonstrates high operational stability of the h-Cr_2_Se_3_ modified SPCE in the detection of 4-NP. To evaluate the repeatability of the sensor, a single h-Cr_2_Se_3_ modified SPCE was used in five set of pH 7 containing 476 µM of 4-NP by CV. In the same manner, five independently prepared h-Cr_2_Se_3_ modified SPCEs were used for the detection of single sample containing 476 µM of 4-NP. Other experimental condtions are similar to Fig. 5A. The h-Cr_2_Se_3_ modified SPCEs demonstrate a relative standard deviation (RSD) of approximately 2.2%, and show appropriate repeatability in the detection of 4-NP. In addition, the RSD about 2.1% was observed for five independently prepared h-Cr_2_Se_3_ modified SPCEs towards detection of 4-NP. The result also demonstrates good reproducibility of the sensor matrix.

#### Real sample analysis

The practicality of amperometric analysis employing the h-Cr_2_Se_3_ modified SPCE was verified using 4-NP spiked tap water and river water samples. Contaminant-free water samples were used as collected, with no treatment prior to analysis, and known concentrations of 4-NP added at pH7. Recovery was calculated using the standard addition method. The 4-NP concentrations determined for spiked tap and river water samples are tabulated in Table 2. The obtained recovery values range from 97.9 to 98.8%, with an average relative standard deviation of 2.9%. These results confirm the practical application of h-Cr_2_Se_3_ modified SPCE for the determination of 4-NP in water samples.

**Table 2:** Determination of 4-NP in different water samples using h-Cr_2_Se_3_ modified SPCE. RSD is related to 3 measurements.

**Sample**	**Added (µM)**	**Found (µM)**	**Recovery (%)**	**RSD (%)**
Tap water	0	Not detected	Not detected	—
	0.05	0.047	94	2.1
	0.1	0.101	101	2.6
	0.15	0.148	98.6	2.3
River water	0	Not detected	Not detected	—
	0.05	0.052	104	3.5
	0.1	0.095	95	3.2
	0.15	0.146	97.3	3.4

## Conclusions

In summary, we have synthesized h-Cr_2_Se_3_ using a simple hydrothermal method and employed it as an electrode material for the first time in the sensitive detection of 4-NP. The physicochemical characterizations confirm the presence of pure h-Cr_2_Se_3_. The h-Cr_2_Se_3_ modified SPCE demonstrated significant advantages over previously reported 4-NP sensors, such as low LOD, wider linear response range, and high detection sensitivity. In addition, the h-Cr_2_Se_3_ modified electrode exhibited a superior capacity for the selective detection of 4-NP. The stability of the sensor is shown to be appropriate for the precise detection of 4-NP in real samples. The sensor also demonstrates highly selective detection of 4-NP in the presence of electrochemically reducible compounds, phenolic compounds and metal ions.

## Experimental

### Materials

Chromium (II) acetate (Cr_2_(CH_3_CO_2_)_4_(H_2_O)_2_), selenium powder (Se), and 4-nitrophenol were purchased from sigma Aldrich. Hydrazine hydrate was purchased from Acros Oroganics. The supporting electrolyte was prepared using 0.05 M Na_2_HPO_4_ and NaH_2_PO_4_ in double distilled water, and adjusted to pH7 through addition of NaOH or H_2_SO_4_. Analytical grade reagents were used without further purification. Screen-printed carbon electrodes were purchased from Zensor R&D Co., Ltd., Taipei, Taiwan.

### Hydrothermal synthesis of h- Cr_2_Se_3_ and electrode modifications

A simple hydrothermal method was used for the synthesis of h- Cr_2_Se_3_. In brief, a suspension of 20 mM chromium acetate and 80 mM selenium was prepared in 25 mL of double distilled water. 6 mL of hydrazine hydrate was gradually added to this suspension under continuous stirring. After 30 min, the mixed solution was transferred to a Teflon sealed auto clave and heated to 180 °C for 16 h. The obtained product (h- Cr_2_Se_3_) was cooled at room temperature, washed with double distilled water and ethanol, and dried in an air oven for 12 h.

The h-Cr_2_Se_3_ dispersion was prepared by the addition of 1 mg of h-Cr_2_Se_3_ to 1 mL of ethanol, and mixing by sonication for 15 minutes. Electrode modification consisted of 6 µL h-Cr_2_Se_3_ suspension drop-coated onto the SPCE surface, and oven drying of the electrode at 45 °C. This h-Cr_2_Se_3_ modified SPCE was used for all reported electrochemical detection of 4-NP.

### Characterization techniques

Scanning Electron Microscopy (SEM) was performed using Hitachi S-3000 H electron microscope. Energy dispersive X-ray (EDX) spectrum was recorded using HORIBA EMAX X-ACT attached with Hitachi S-3000 H scanning electron microscope. XRD characterization was carried out using XPERT-3 diffractometer with Cu Kα radiation (K = 1.54 Å). Electrochemical impedance spectroscopy (EIS) was performed using IM6ex ZAHNER impedance measurement unit. CV and amperometric studies were performed using CHI611A electrochemical analyzer. Conventional three-electrode system was used for electrochemical studies. The modified SPCE was used as a working electrode, and saturated Ag/AgCl and platinum electrodes were used as the reference and auxiliary electrodes respectively. CV experiments were performed in a potential range from 0.4 to −0.7 V at a scan rate of 50 mV s^−1^. All electrochemical measurements were carried out at a room temperature in N_2_ saturated electrolyte solution at pH 7.

## Electronic supplementary material

Supplementary Information
