# Supplementary material for: Hydrothermal Synthesis of Cr2Se3 Hexagons for Sensitive and Low-level Detection of 4-Nitrophenol in Water
Source: Sci Rep. 2018 Mar 19;8:4839. doi: 10.1038/s41598-018-23243-3 (PMC5859153; doi:10.1038/s41598-018-23243-3)
Supplement: Supplementary file 1 — Supplementary Information [file 41598_2018_23243_MOESM1_ESM.pdf]

Hydrothermal Synthesis of Cr_2Se_3 Hexagons for Sensitive and Low-level Detection of 4-Nitrophenol in Water

Sukanya Ramaraj[1], Sakthivel Mani[1], Shen-Ming Chen[1]*, Selvakumar Palanisamy[1, 2], Vijayalakshmi Velusamy[2]**, James M. Hall[2], Tse-Wei Chen[1], Tien-Wei Tseng[1]

[1]Electroanalysis and Bioelectrochemistry Lab, Department of Chemical Engineering and Biotechnology, National Taipei University of Technology, Taipei, Republic of China

[2]Division of Electrical and Electronic Engineering, School of Engineering, Manchester Metropolitan University, Manchester, UK

Correspondence and requests for materials should be addressed to S.M.C (smchen78@ms15.hinet.net) and V.V (V.Velusamy@mmu.ac.uk)

Supporting information

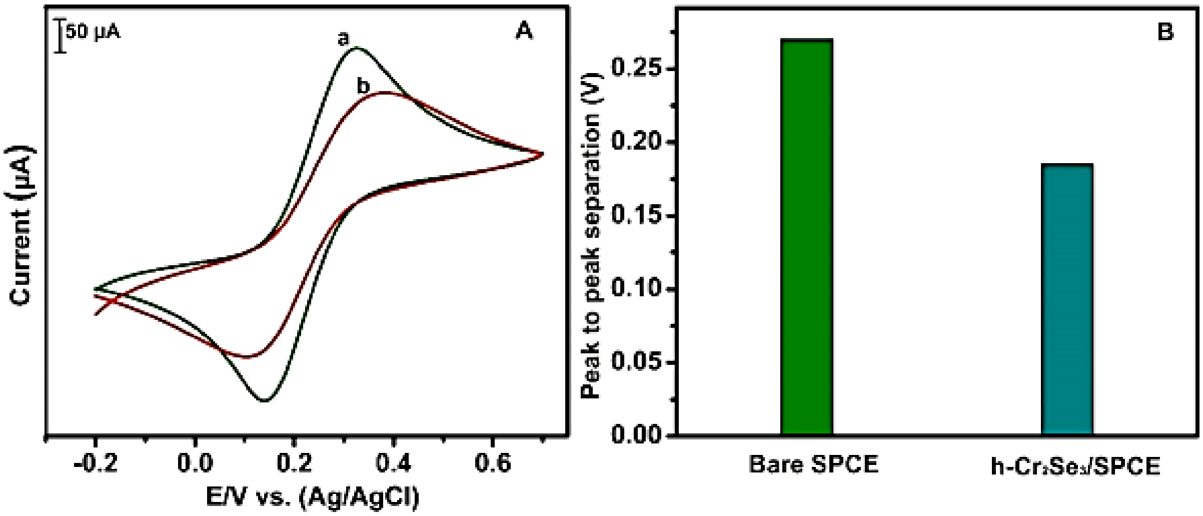

Fig. S1 (A) CV response of h-Cr_2Se_3/SPCE (a) and bare SPCE (b) in 5 mM of $[Fe(CN)6]^{3-/4-}$ containing 0.1 M of KCl at a scan rate of 50 mV/s. B) Corresponding bar graph for peak-to-peak separation of $[Fe(CN)6]^{3-/4-}$ at h-Cr_2Se_3/SPCE and bare SPCE.

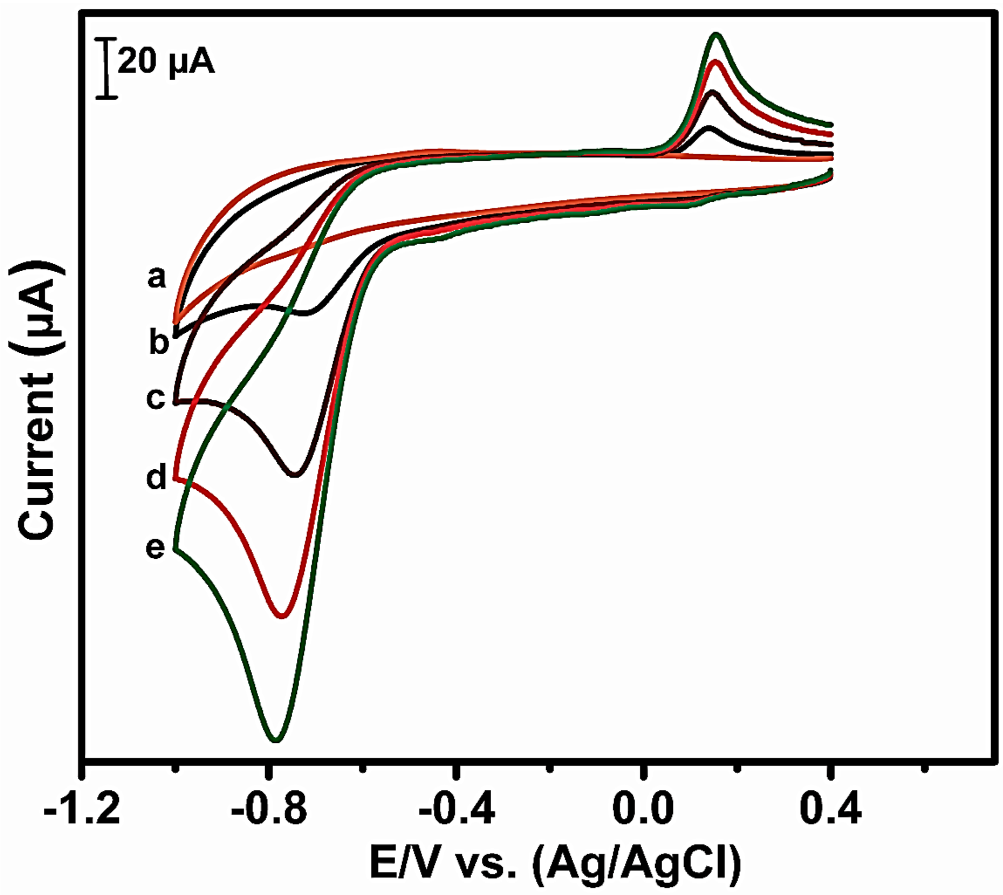

Fig. S2 CV response of h-Cr_2Se_3 modified SPCE for the absence (a) and presence of 100 (b), 380 (c), 650 (d) and 909 μM (e) 4-NP at pH 7 at a scan rate of 50 mVs^{-1}

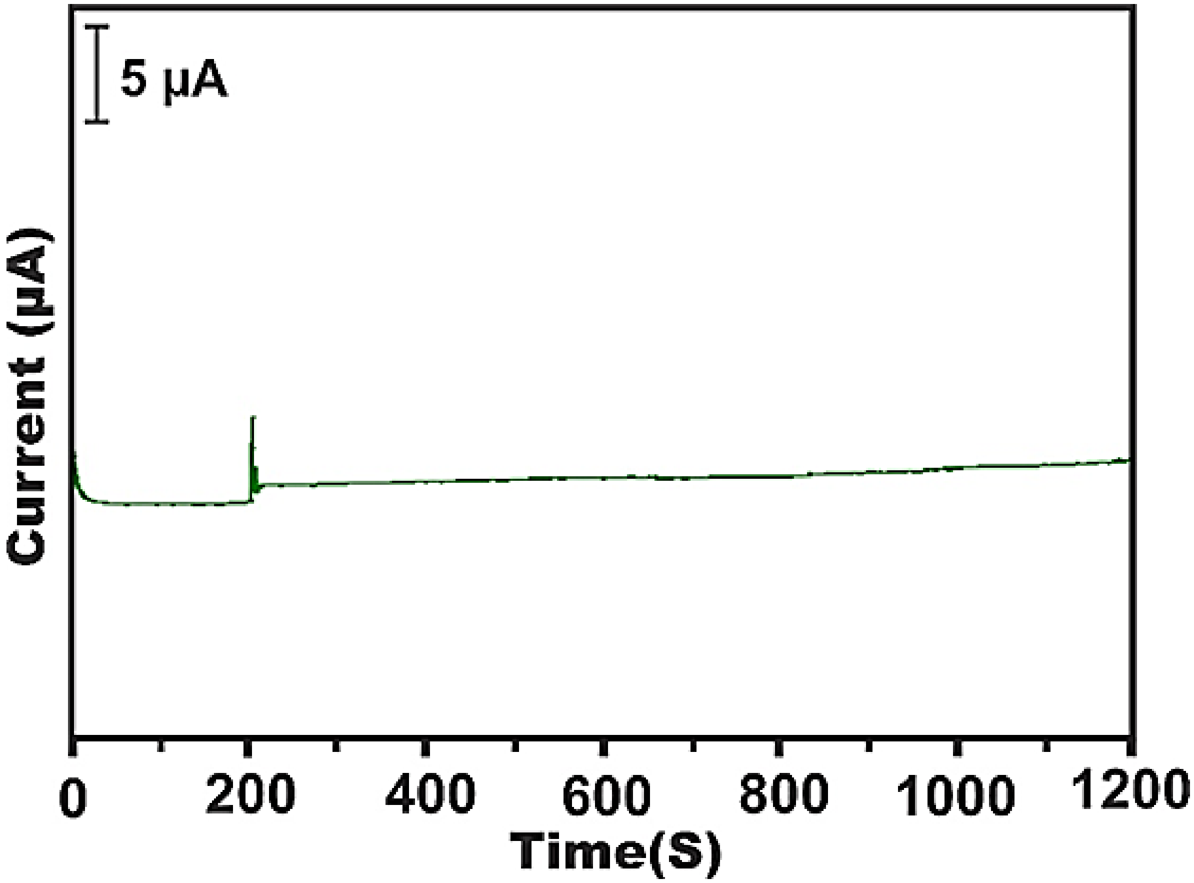

Fig. S3 Amperometric i-t response of h-Cr_2Se_3 modified SPCE for the addition of 10 µM 4-NP into the constantly stirred pH 7, and its background current response for 1200 s. Applied potential = -0.73 V.
